# *p*-Sulfonato-Calix[4]arene Micelles Stabilize a Povidone Iodine Solution: Supramolecular Interactions, Iodine Retention, and Bactericidal Activity

**DOI:** 10.3390/nano13020286

**Published:** 2023-01-10

**Authors:** Rossella Migliore, Loredana Ferreri, Danilo Aleo, Claudia Giovanna Leotta, Giovanni Mario Pitari, Nicola D’Antona, Carmelo Sgarlata, Grazia Maria Letizia Consoli

**Affiliations:** 1 Institute of Biomolecular Chemistry—National Research Council (C.N.R.), Via Paolo Gaifami 18, 95126 Catania, Italy; 2 MEDIVIS Srl, Via Carnazza 34C, Tremestieri Etneo, 95030 Catania, Italy; 3 Vera Salus Ricerca, Via Sigmund Freud 62/B, 96100 Siracusa, Italy; 4 Department of Chemical Sciences, University of Catania, Viale Andrea Doria 6, 95125 Catania, Italy

**Keywords:** povidone-iodine, sulfonato-calix[4]arene, micellar aggregate, non-covalent interactions, antibacterial activity

## Abstract

Povidone iodine (PVPI) is an antiseptic widely used against a broad spectrum of pathogens. However, undesired side-effects are still associated with PVPI treatment due to the irritant effect of iodine. Reducing the concentration of a PVPI formulation could provide safer and more friendly formulations, for routine use and applications in very delicate organs such as the eye. However, managing the storage of a low-concentration solution of PVPI is challenging due to the high iodine volatility. In this study, we demonstrated that an amphiphilic *p*-sulfonato-calix[4]arene derivative forming micelles (SC4OC6) improves the stability of a 0.1% PVPI aqueous buffered solution. UV-vis and NMR spectra as well as dynamic and electrophoretic light scattering measurements showed that SC4OC6 establishes non-covalent supramolecular interactions with PVPI, resulting in the formation of nanoaggregates with a negatively charged surface. Isothermal titration calorimetry provided the aggregation parameters and evidenced that the formation of the supramolecular assembly is an enthalpically favored process. The interaction of SC4OC6 with PVPI enhances the iodine retention and stability of the solution without affecting the rapid and effective bactericidal activity of PVPI, as demonstrated by a time-killing assay with *Staphylococcus epidermidis*.

## 1. Introduction

Iodine is a broad-spectrum antiseptic displaying high efficiency and low cost. The antimicrobial action of iodine is rapid, even at low concentrations, but the exact mode of action is still unknown. It has been proposed that iodine penetrates the cell wall of microorganisms and attacks key groups of proteins, nucleotides, and fatty acids ensuring the rapid death of pathogens [1,2]. However, iodine is not stable, easily sublimates and decomposes, and is difficult to store. Increasing the iodine concentration could enhance the storage time but high doses of elemental iodine are responsible for irritation, pain, burning, systemic cytotoxicity, and bioaccumulation. To prepare safer and more stable formulations, the entrapment of iodine in iodophors has turned out to be a valid strategy. An iodophor enhances iodine water solubility and stability and reduces its toxicity by controlling the iodine concentration through a slow release over a sustained time [3].

Polyvinylpyrrolidone iodine (PVPI) is one of the most used antiseptic iodophors in clinical applications [4,5]. As high concentrations of iodine are responsible for undesired side-effects, solutions more diluted than the commercial PVPI ones (usually 10% concentration) are desirable to achieve safer and more effective formulations. Paradoxically, PVPI exhibits a larger biocide effect when at lower concentrations. This behavior was ascribed to the different aggregation state of PVPI. At 10% concentration, the aggregated polymer entraps iodine more efficiently and the free iodine, which is responsible for the rapid antimicrobial activity, has been determined to be around 1 ppm. By contrast, at a 0.1% concentration, the less aggregated PVPI yields around 20 ppm of free bioactive iodine [6]. Currently, in nosocomial applications, the simple dilution of commercially available PVPI with water or saline has been advocated to produce PVPI in the 0.25–0.5% range; however, it has been demonstrated that such dilution provides a solution with physicochemical features different from solutions specifically manufactured at lower concentration [7]. Recently, PVPI at concentrations below 2% has been proposed for nasal and oral antisepsis in the COVID-19 pandemic [8]. Less concentrated PVPI solutions are very unstable, especially when stored in plastic containers, and therefore, the stabilization of low-concentration PVPI solutions is a topic of great interest though still challenging.

Calix[n]arenes are a family of macrocyclic polyphenolic oligomers relevant in supramolecular chemistry. They possess a hydrophobic cavity able to host ions and neutral molecules and a remarkable synthetic versatility that allows for obtaining a very large number of derivatives for applications in different fields, including pharmaceutical and biomedical areas [9,10]. Among calixarene derivatives, *p*-sulfonato-calix[4]arenes have gained interest in drug discovery and drug delivery [11,12], including antimicrobial applications [13,14], due to their proved low cytotoxicity [15].

It has been reported that simple anionic micelles binding PVPI can favor the iodine-polymer interaction [16]. With this in mind, we decided to investigate whether a polyanionic *p*-sulfonato-calix[4]arene amphiphile (SC4OC6), known to form negatively charged micelles in aqueous media [17], can establish suitable interactions with PVPI and, by changing the aggregation state of the polymer, can improve the stability of a 0.1% PVPI phosphate-citrate buffer solution (pH 6), a medium typically used in pharmaceutical formulations. The self-assembly of SC4OC6 in buffered solution was investigated by dynamic light scattering (DLS) and the aggregation parameters determined by isothermal titration calorimetry (ITC). The formation of the PVPI/SC4OC6 nanoassembly was explored by nuclear magnetic resonance (NMR) spectroscopy, UV-vis spectrophotometry, ITC, DLS, and electrophoretic dynamic scattering (ELS), which provided key information for the development of an efficient PVPI-micellar adduct. The effect of SC4OC6 on the stability of PVPI and the consequent enhanced iodine retention capability were investigated by UV-vis spectrophotometry. Finally, the antibacterial activity of the PVPI/SC4OC6 nanoassembly was evaluated against a *Staphylococcus epidermidis* strain.

## 2. Materials and Methods

### 2.1. Materials

Reagents were purchased from Sigma Aldrich (Milan, Italy), and used as received without further purification. Sodium dodecyl sulfate (SDS) and *p*-sulfonato-calix[4]arene (SC4OH) were purchased from Sigma-Aldrich. PVPI was purchased from Farmalabor Srl. Phosphate/citrate buffer (PCB) at 10 mM concentration was prepared by weighing the proper amounts of phosphate and citrate salts (Sigma-Aldrich). The pH 6 value of the buffer solution was checked potentiometrically. High-purity water (Millipore, Milli-Q Element A 10 ultrapure water) and A grade glassware were employed throughout. The *p*-sulfonato-calix[4]arene derivative (SC4OC6) was synthesized as reported in the literature and characterized by NMR spectroscopy [18]. *Staphylococcus epidermidis* (ATCC^®^ 12228, Origin Strain No. CECT^®^ 231) and all other reagents for the biological assays were obtained from Merck KGaA (Darmstadt, Germany). Bacteria cultures were prepared at 37 °C in 1.5% agar plates supplemented with nutrient broth containing 3% beef extract and 5% peptone.

### 2.2. Preparation of the PVPI/SC4OC6 Assembly

A 0.1% solution of PVPI, prepared by dissolving 12 mg of PVPI in 12 mL of PCB, was added to different amounts of solid SC4OC6 to obtain clear solutions having different concentrations (0.25, 0.5, and 1 mg/mL).

### 2.3. Preparation of the SC4OC6/Iodine Complex

A 0.6% solution of SC4OC6 (24 mg) was added to 2.4 mg of iodine in 4 mL of PCB and the mixture was stirred up to the total dissolution of iodine. 

### 2.4. Characterization of the PVPI/SC4OC6 Assembly

UV-vis spectra of PCB solutions of 0.1% PVPI alone and with different amounts of SC4OC6 (0.25, 0.5, and 1 mg/mL) were recorded on an Agilent Technologies 8453 UV−vis spectrophotometer.

^1^H-NMR (400.13 MHz, D_2_O, 297 K) spectra of SC4OC6 (1 mg/mL), 0.1% PVPI, and 0.1% PVPI/SC4OC6 (1:1, *w*:*w*) were acquired on a Bruker Avance 400 spectrometer. Chemical shifts (δ) are expressed in parts per million (ppm) and reported relative to the residual water proton peak. SC4OC6: δ 0.83 (br s, 12 H, 4 × CH_3_), 1.29 (br s, 24 H, 12 × CH_2_), 1.89 (br s, 8 H, 4 × CH_2_), 3.31 and 4.35 (br AX system, 8 H, 4 × ArCH_2_Ar), 3.82 (t, 8 H, 4 × OCH_2_), 7.21 (s, 8 H, 4 × ArH). PVPI: δ 1.3–1.8 (–CH_2_–CH– methylene main chain), 1.8–2.1 (–CH_2_–CH_2_–CH_2_), 2.1–2.5 (–CH_2_–C=O), 3.0–3.4 (–CH_2_– methylene side chain), 3.4–3.9 (–CH_2_–CH– methine main chain). PVPI/SC4OC6: δ 0.97 (br s, 12 H, 4 × CH_3_), 1.41 (br s, 24 H, 12 × CH_2_), 1.92 (br s, 8 H, 4 × CH_2_), 1.8–2.1 (–CH_2_–CH_2_–CH_2_), 2.1–2.5 (–CH_2_–C=O), 3.0–3.4 (–CH_2_– methylene side chain), 3.31 and 4.35 (br AX system, 8 H, 4 × ArCH_2_Ar), 3.4–3.9 (–CH_2_–CH– methine main chain), 3.88 (t, 8 H, 4 × OCH_2_), 7.24 (s, 8 H, 4 × ArH).

### 2.5. Size and Zeta Potential Measurements 

The measurements were carried out on samples of SC4OC6 (1 mg/mL), 0.1% PVPI, and 0.1% PVPI/SC4OC6 (1 mg/mL) in PCB by using a ZetaSizer NanoZS90 (Malvern Instrument, Malvern, UK), equipped with a 633 nm laser, at the scattering angle of 90° and at 25 °C temperature. The size of the particles was calculated from the diffusion coefficient by using the Stokes–Einstein equation:

$$D=\frac{kT}{6\pi \eta R}$$

where *D* is the diffusion coefficient, *k* is the Boltzmann constant, *T* is the absolute temperature, *η* is the solvent viscosity, and *R* is the solute radius.

The zeta potential (*ζ*) was calculated by using Henry’s equation:

$$UE=\frac{2\varepsilon \zeta }{3\eta }\;f\;\left(Ka\right)$$

where *UE* is the electrophoretic mobility, *ε* is the dielectric constant, *f* (*Ka*) is the Henry’s function, and *η* is the viscosity.

### 2.6. Isothermal Titration Calorimetry

ITC experiments were run at 25 °C by using a nano-isothermal titration calorimeter Nano-ITC (TA Instruments, New Castle, DE, USA) with an active cell volume of 0.988 mL and a 250 µL injection syringe. During the titration, the reaction mixture in the cell was stirred at 250 rpm. Measurements were carried out in the overfilled mode [19], which does not require any correction for liquid evaporation and for the presence of the vapor phase. The power curve was integrated using the NanoAnalyze software (TA Instruments, New Castle, DE, USA) to obtain the gross heat evolved/absorbed in the reaction. The calorimeter was calibrated chemically through the procedure previously described [20]. An electrical calibration was also carried out.

Self-aggregation experiments

Two sets of measurements were conducted, i.e., (1) the titration of concentrated surfactant solutions into a buffer solution or (2) the titration of the polymer solution into either a buffer or a surfactant solution. The first set of experiments [21] was run to obtain accurate values for both the critical micellar/aggregation concentration (CMC/CAC) and the enthalpy of micellization (Δ*H_mic_*) for the amphiphilic calixarene SC4OC6 and sodium dodecyl sulphate under the experimental conditions employed in the present work. The second set was run to study the self-aggregation process of PVPI in the presence and absence of the calixarene-based micelles. In both cases, the analysis of the heat values recorded in the calorimetric experiments allowed for the direct determination of the CMC/CAC and Δ*H_mic_* values.

PVPI/SC4OC6 micelle interaction

Proper ITC titrations were also carried out to determine the thermodynamic parameters that drive the binding interactions between PVPI and the surfactants in solution. In this case, the net heats of reaction to be analyzed were obtained by subtracting the heat evolved/absorbed in the blank experiment (which consisted of a titration of the polymer solution into a solution containing only buffer) from the raw heats. The net heats of reaction were treated by HypCal [22], a software that allows for the determination of the equilibrium constants and enthalpy changes for the formation of complex species in solution by a non-linear least-squares minimization of the function:

$$U=\sum^{\;}{\left(Q_{obs.\;}-Q_{calc.}\right)}^{2}$$

where *Q_obs._* is the observed net heat for a given reaction step, while *Q_calc._* is calculated as

$$Q_{calc.}=-\sum^{\;}\delta n\Delta H$$

where δ*n* is the change in the number of moles of a reaction product and Δ*H* is the molar formation enthalpy of the reaction product. The sum is carried out over all the reaction steps; the squared residuals ${\left(Q_{obs.\;}-Q_{calc.}\right)}^{2}$ are summed over all the titration points. Log*K* values and thermodynamic parameters were obtained by analyzing simultaneously calorimetric data obtained from different titrations.

### 2.7. Evaluation of the Amount of Molecular Iodine in Solution by Cyclohexane Extraction

An amount of 1 mL of PVPI/SC4OC6-buffered solution was manually shaken (20 s) with 1 mL of cyclohexane (partial extraction) or extracted by cyclohexane (1 mL × 3, exhaustive extraction). The amount of iodine dissolved in cyclohexane was determined by UV-vis spectrophotometry using a calibration curve.

### 2.8. Diffusion of Iodine from the Buffered Solution to Cyclohexane 

The buffered solutions of 0.1% PVPI alone or with SC4OC6 (0.25 mg/mL) were placed in vials (1 mL each) and then cyclohexane (1 mL) was laid on each sample. Cyclohexane was collected from each couple of samples (PVPI with and without SC4OC6) at four different times (10, 20, 30, and 60 min), and the amount of iodine dissolved in the organic solvent was determined by UV-vis spectrophotometry using a calibration curve. The experiments were performed in triplicate and repeated three times with similar results.

### 2.9. Stability Study

Samples of 0.1% PVPI alone and with SC4OC6 at different concentrations (0.25, 0.5, and 1 mg/mL) were stored in glass or plastic containers at 25 and 4 °C. The amount of iodine in each sample was determined after a time by exhaustive extraction with cyclohexane. 

### 2.10. Time-Kill Assay

Bacterial suspensions were prepared from 16 h growth cultures diluted to ~1.5 × 10^8^ CFU/mL, as estimated by comparison with a 0.5 McFarland turbidity standard employing an UV spectrophotometer (Synergy HT, BioTek Agilent, Santa Clara, CA, USA). The final assay concentration of 6 × 10^5^ CFU/mL of *Staphylococcus epidermidis* was achieved through intermediate bacterial suspensions of ~7.5 × 10^6^ CFU/mL. Subsequently, 0.1 mL of bacterial suspensions was added to 1.9 mL of each sample solution (CTR, SC4OC6, PVPI, and PVPI/SC4OC6) and incubated for different times (10, 20, and 40 s, and 1, 2, 4, 8, and 60 min) before residual iodine neutralization with a 0.5% sodium thiosulfate solution (1:10 dilution, *v*/*v*). After two additional rounds of dilutions (1:10, *v*/*v*) in sterile saline solutions, 0.1 mL of each sample was plated (by spreading) onto an agar-enriched culture medium and incubated at 37 °C (24 h) before colony counting.

Statistical Analysis: Results are means ± SEM of three independent experiments performed in duplicate. Statistical analyses were performed with two-way ANOVA. *p* values were considered significant at α ≤ 0.05. All analyses were carried out with GraphPad Prism 9.3.1 (GraphPad Software, Inc., San Diego, CA, USA).

## 3. Results and Discussion 

### 3.1. Self-Assembly of SC4OC6 in Buffered Phosphate-Citrate Solution

**Figure 1:**
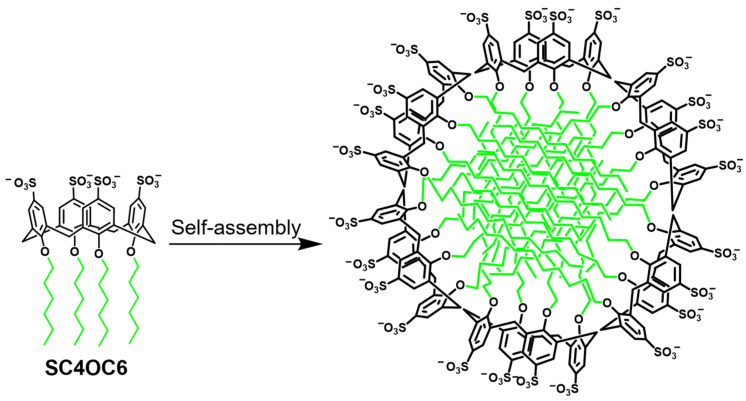
Schematic representation of the SC4OC6 structure and its self-assembling in a micellar aggregate.

The *p*-sulfonato-calix[4]arene derivative SC4OC6, bearing four sulfonate groups and four C6 alkyl chains tethered at the host aromatic rings and hydroxyl groups, respectively, (Figure 1) was synthesized as reported in the literature [18].

The self-aggregation process of the amphiphilic SC4OC6 has already been studied in plain water and in buffered solution [12,23]. However, as the change in the ionic medium can induce a significant variation in the CMC value [24,25,26,27], the aggregation features of SC4OC6 were specifically determined at the experimental conditions employed in the present work (10 mM phosphate/citrate buffer, pH 6, hereinafter referred to as PCB). For comparison, the aggregation of SDS was also examined [28]. Typical calorimetric patterns obtained for these systems are shown in Figures S1 and S2 and the corresponding CMC and ∆*H_mic_* values are reported in Table S1 from the Supplementary Materials.

As previously described in the presence of phosphate buffer [12], the CMC of SC4OC6 results as significantly lower (0.05 mM) if compared to that in plain water (0.49 mM), as expected for amphiphilic molecules in the presence of salts [29,30]. Furthermore, the self-assembly process is less exothermic than in the buffer-free aqueous solutions [31,32,33]. The counterion adsorption causes a decrease in the overall surface charge of the micelles, making the self-aggregation process more favored, i.e., occurring at a lower CMC value. A similar behavior is observed for the SDS micellization process in the presence of a buffering medium (see Table S1). The formation of SC4OC6 nanoaggregates in PCB was confirmed by dynamic light scattering measurements (Figure S3).

### 3.2. Formation and Characterization of the PVPI/SC4OC6 Assembly

**Figure 2:**
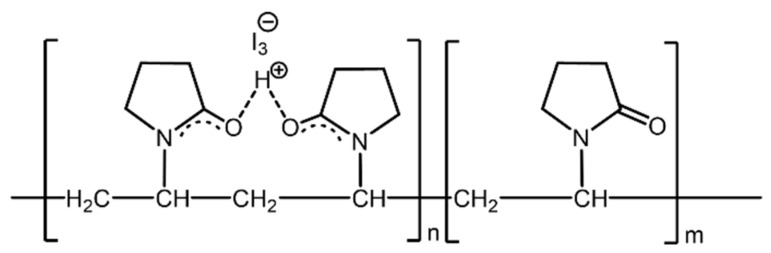
Schematic representation of the structure of the PVPI complex.

Despite the use for over 60 years, the structure of the PVP/iodine complex and the pathway of iodine release are not fully understood. The interaction between PVP and iodine is somewhat controversial and different structures and mechanisms have been proposed. Among them, Xu reported that the H^+^ ion forms a hydrogen bond between the carbonyl groups of two adjacent pyrrolidone rings and, consequently, the triiodide anion (I_3_^−^), which is a “smart” reservoir of molecular iodine, is bound to the resulting cation through electrostatic interaction (Figure 2) [34].

It has been reported that PVP interacts with iodine molecules through halogen bonds. The halogen bond energy as low as 2–8 kcal/mol enables the easy release of iodine [35], which is important for the biocide activity but detrimental for the stability of PVPI solutions due the high volatility of the uncomplexed iodine. With this in mind, we planned to investigate the effect of the SC4OC6 micelles on the stability of a 0.1% PVPI solution.

The polyanionic SC4OC6 micellar aggregate could establish non-covalent interactions with PVPI, including electrostatic interactions with the PVPI residual positive charge. The SC4OC6/PVPI supramolecular nanoassembly could affect the aggregation state of the polymer and slow down the iodine release. The iodine retention could also be favored by halogen bond formation with the electron-rich moieties of SC4OC6 (i.e., aromatic rings, and O and S atoms).

**Figure 3:**
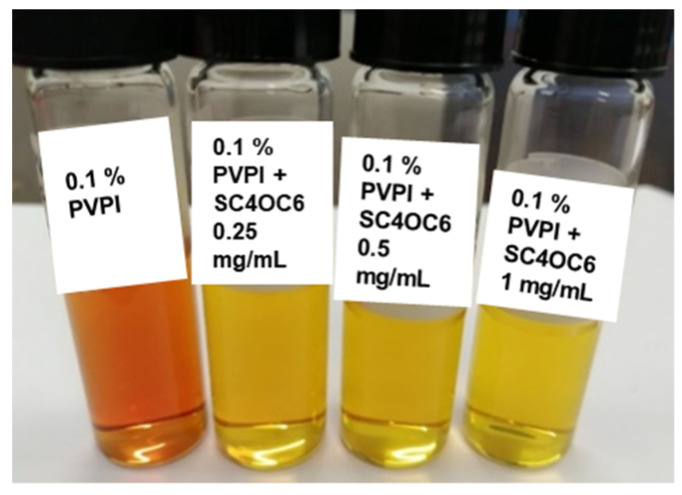
Pictures of 0.1% PVPI solutions alone and with different amounts of SC4OC6 (0.25, 0.5, and 1 mg/mL) in PCB.

The addition of SC4OC6 (0.25, 0.5, or 1 mg/mL) to a 0.1% PVPI solution in PCB determined an immediate change in the color of the PVPI solution, visible to the naked eye (Figure 3).

#### 3.2.1. UV-vis Absorption Spectra Analysis

**Figure 4:**
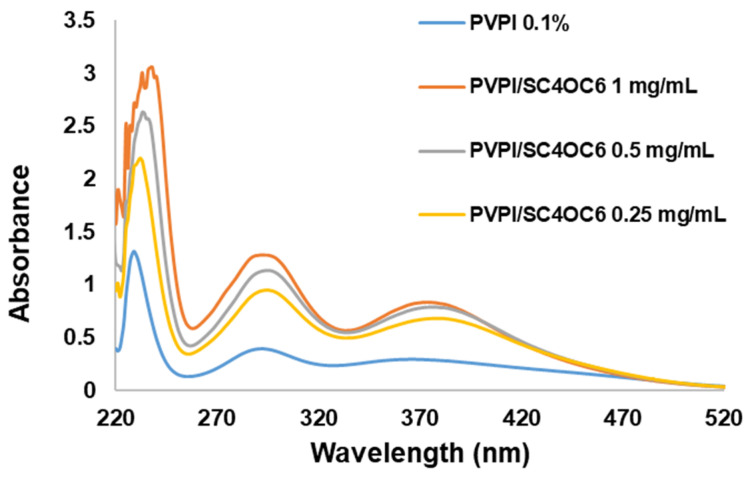
UV-vis spectra of 0.1% PVPI solutions alone and with SC4OC6 (0.25, 0.5, and 1 mg/mL) in PCB.

UV-vis absorption spectra of the solutions confirmed that SC4OC6 establishes interactions with PVPI. In line with the literature, UV-vis spectra of PVPI showed one band at 229 nm (I^−^) and two distinct bands centered at 290 and 359 nm ascribable to I_3_^−^ (iodate and iodine oxyanion are transparent at λ > 300 nm). The broad band with maximum absorption at 359 nm includes the elemental iodine (I_2_) absorption (Figure 4). 

The interaction of SC4OC6 with PVPI determined a bathochromic and hyperchromic effect: the absorption of PVPI at 290 nm and 359 nm was shifted to 295 nm and 379 or 382 nm, respectively, depending on the concentration of SC4OC6; the hyperchromic effect depended on the amount of SC4OC6 added.

The UV-Vis spectrum of the PVPI/SC4OC6 assembly was quite similar to that of PVPI with sodium dodecyl sulfate, despite the smaller SC4OC6 concentration employed to form the aggregate (Figure S4). The micelles of SDS were reported to favor the interaction of iodine with the polyvinyl pyrrolidone polymer, and the observed bathochromic effect was associated with the interaction of the anionic micelles with the positive centers on the pyrrolidone group [16].

#### 3.2.2. NMR Analysis

**Figure 5:**
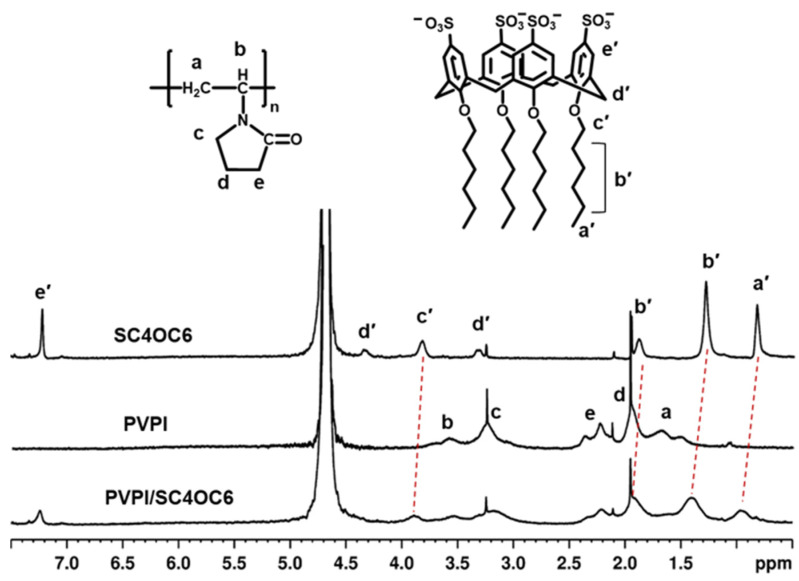
^1^H NMR spectra (400 MHz, D_2_O, 297 K) of SC4OC6, PVPI, and PVPI/SC4OC6.

The interaction of PVPI (1 mg/mL) with SC4OC6 (1 mg/mL) was also corroborated by ^1^H NMR spectra (Figure 5). The proton spectra showed a downfield shift in the SC4OC6 resonances (deshielding effect, Δδ 0.03, 0.06, 0.12, and 0.13 ppm for ArH, OCH_2_, CH_2_, and CH_3_, respectively), and a small upfield shift in the detectable PVPI resonances (Δδ 0.02–0.03 ppm). The observed shifts suggested that, upon PVPI-SC4OC6 binding, the methylene and methyl groups of the calixarene alkyl pendants experience a more polar framework than when in the unbound micelle, whereas PVPI undergoes a more hydrophobic environment such as that provided by the SC4OC6 micellar backbone.

#### 3.2.3. Size and Zeta-Potential Measurements

PVPI in solution exists as aggregates in which the iodine molecules become adsorbed on the assembly surface and enter the aggregate inner spaces where iodine is converted into polyiodides [34,35]. To investigate the effect of SC4OC6 on the PVPI aggregation state, we performed dynamic light scattering measurements. These experiments showed that a 0.1% PVPI solution in PCB contains nanoaggregates with a mean hydrodynamic diameter of 14.2 nm (Z average), I% = 16.1 nm, and polydispersity index (PDI) of 0.26 (Figure S5a). The DLS analysis of the PVPI/SC4OC6 (0.25 mg/mL) assembly showed the presence of aggregates with a slightly larger mean hydrodynamic diameter and higher PDI (Z average = 17.7 nm, I% = 21.6 nm, and PDI of 0.4) (Figure S5b). A more significant variation was instead observed for zeta potential, which changed from −5.4 mV (PVPI alone) to −13.9 mV for PVPI in the presence of SC4OC6 (0.25 mg/mL), suggesting a rearrangement of the polymer around the anionic micelles.

#### 3.2.4. ITC Measurements

For charged surfactant–neutral polymer systems, two critical concentrations, namely critical aggregation concentration (CAC) and polymer saturation point concentration (PSP), are proposed [36]. The first one is the concentration from which surfactant molecules begin to interact with the polymer chains, while the second parameter is the concentration at which the polymer chains become saturated by micelle-like surfactant aggregates. When the surfactant concentration is below CAC, there is no interaction between surfactant molecules and polymer chains; consequently, when the surfactant concentrations range from CAC to PSP, micelle-like surfactant aggregates interact with the polymer chains mainly by hydrophobic interactions. Finally, when the surfactant concentration is above the PSP, the interactions are saturated and free micelles are formed [16].

Our main interest was the comprehension of the effect of the micelles on the PVPI properties as they may interfere in the release of iodine. In particular, the SC4OC6 micelles may reduce the critical aggregation concentration of PVPI by effectively interacting with the polymeric chains. For this reason, to reproduce the conditions at which the interaction between the polymer and micelles may occur, we decided to titrate the polymer solution into SC4OC6 micellar solutions. 

**Figure 6:**
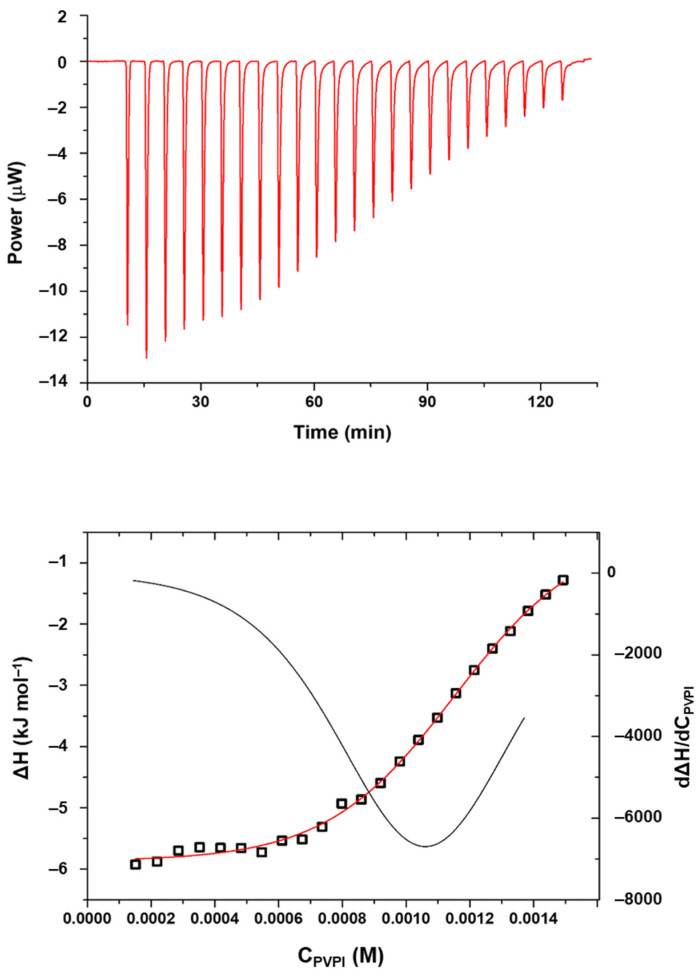
ITC titration of a 2.5 mg/mL PVPI solution into PCB at 25 °C (**top**); enthalpy of reaction as a function of the total concentration of PVPI in the calorimetric cell (C_PVPI_ is calculated as the monomer unit; squares: enthalpy values, red line: curve fitting, black line: first derivative) (**bottom**).

As PVPI self-aggregates in solution, its critical aggregation concentration (CAC) was determined as well as the enthalpy value associated with this process in PCB (Table 1) by means of calorimetric measurements. Figure 6 shows an example of a calorimetric titration.

**Figure 7:**
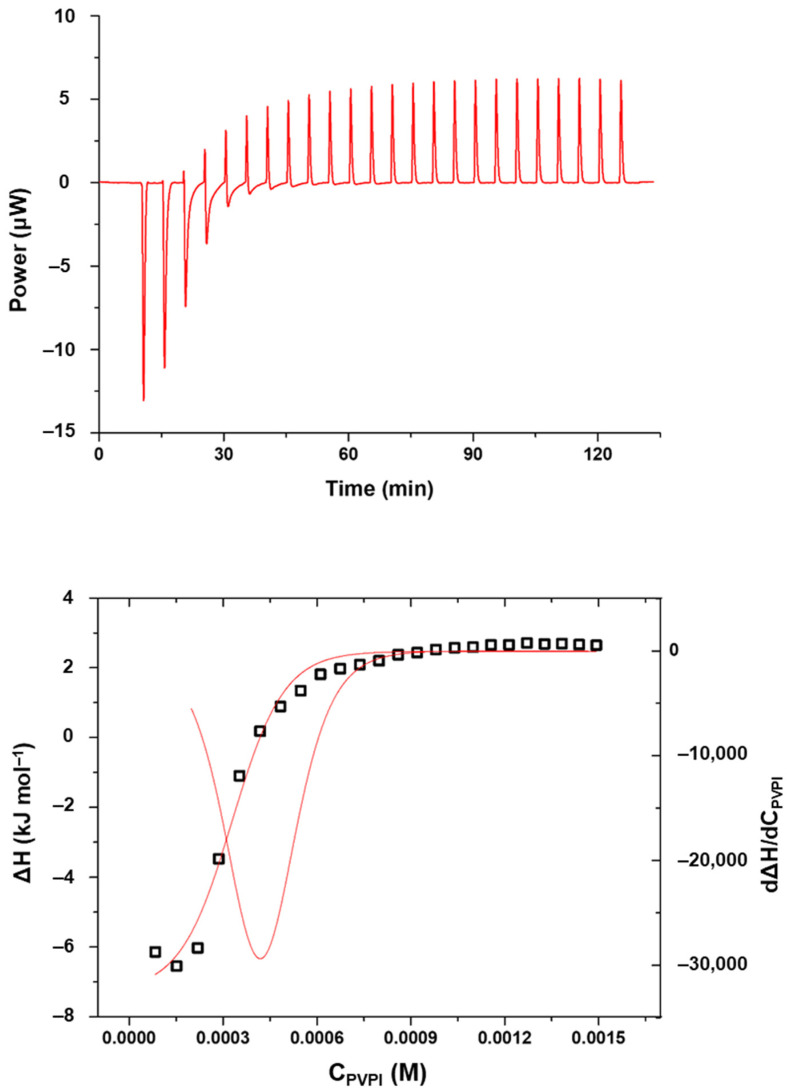
ITC titration of a 2.5 mg/mL PVPI solution into SC4OC6 1 mM in PCB at 25 °C (**top**); enthalpy of reaction as a function of the total concentration of PVPI in the calorimetric cell (C_PVPI_ is calculated as the monomer unit; squares: enthalpy values, red lines: curve fitting and first derivative) (**bottom**).

Once the conditions for the formation of PVPI aggregates were established, the process was also studied in the presence of SC4OC6 micellar aggregates, to establish to what extent the presence of the micelles promotes the self-aggregation of the polymer. In a typical calorimetric experiment, a solution of PVPI was titrated into a solution containing SC4OC6 micellar aggregates (Figure 7).

To evaluate the effect of the alkyl chains and the micellar structure of SC4OC6 on the polymer aggregation process, the same calorimetric experiments were carried out in the presence of the non-amphiphilic *p*-sulfonato-calix[4]arene (SC4OH), which does not bear alkyl chains tethered to the calixarene phenolic OH groups, and is not able to form micelles (Figure S6). Moreover, to establish the role of both the macrocyclic platform and the aliphatic tails, the experiments were performed also using the commercial surfactant sodium dodecyl sulfate (SDS, Figure S7). For all the systems examined, the values of both the critical aggregation concentration and enthalpy of aggregation of PVPI were determined and are shown in Table 1.

The values reported in Table 1 show that the presence of micellar aggregates, formed by the amphiphilic calixarene SC4OC6 or by the commercial surfactant SDS, causes a significant reduction in the critical aggregation concentration of PVPI. On the other hand, the presence of the non-amphiphilic SC4OH macrocycle does not promote the PVPI aggregation process, making it even impossible to determine the thermodynamic parameters.

Concerning the enthalpy values, although the CAC values are comparable in the presence of both the micellar aggregates based on SC4OC6 and SDS molecules, the Δ*H* contribution is doubled in the case of micelles formed by the calixarene, indicating that in the case of SC4OC6, the process takes place with a greater enthalpy gain.

Furthermore, it may be observed that the reaction in the presence of SC4OC6-based aggregates becomes exothermic as the titration proceeds (Figure 7), revealing that an interaction between PVPI and SC4OC6 micelles is effectively occurring in the calorimetric cell. It should be noted that this phenomenon is not observed in the presence of micelles formed by SDS, where the amount of heat recorded after the self-aggregation of PVPI is negligible (Figure S7). This result highlights the advantage of using the amphiphilic SC4OC6 as, in addition to promoting the aggregation of PVPI at lower concentrations, it can efficiently interact with the polymer.

The ITC experiments also enabled the quantitative determination of the entity of the binding process by properly fitting the calorimetric data. The binding of SC4OC6 micelles to PVPI was analyzed with HypCal using a 2:1 binding model, in line with the “sequential binding model” often described in the literature for similar systems [37]. The cumulative binding constant was found to be 5.5(1) log units. The binding process resulted as enthalpically driven and favored with a small favorable entropic contribution. The enthalpy change was –29.17(5) kJ mol^−1^ while the Δ*S* value was +7.5(1) J mol^−1^ K^−1^ (parameters are displayed in Figure S8).

The (small) favorable entropic contribution is due to desolvation of both the polymer chain and the micelle surface upon binding. This result suggests that the polymer interacts with the exterior surface of the micelles and such interactions cause the release of superficial water of hydration to the bulk solvent [12,38,39]. The favorable Δ*H* value is likely due to CH-π and electrostatic interactions between the calixarene hydrophilic heads and the PVPI backbone that advantageously balance the enthalpic cost for desolvation [40,41].

### 3.3. Stabilizing Effect of SC4OC6 on the 0.1% PVPI Solution

It is known that a higher aggregation state of PVPI reduces the amount of volatile free iodine in solution [6]. Analogously, the interaction of SC4OC6 with PVPI may change the aggregation state of the polymer and determine a more efficient entrapment of volatile iodine with a consequent enhancement of the solution stability.

The effect of SC4OC6 on the stability of the 0.1% PVPI solution was investigated by monitoring over time the absorption of the triiodide bands of the complex in comparison to those of PVPI alone. The samples stored in glass containers and in the dark were satisfactorily stable at room temperature and at 4 °C storage. After 3 months, about 98% of the triiodide absorption was retained, as assessed by the UV-vis spectra of PVPI with and without SC4OC6. The effect of the calixarene on iodine retention was clearly evident when the samples were heated at 60 °C for 24 h. The retained triiodide (absorbance at 295 nm) was 7% for PVPI alone and 58% in the complex with SC4OC6 (1 mg/mL).

**Figure 8:**
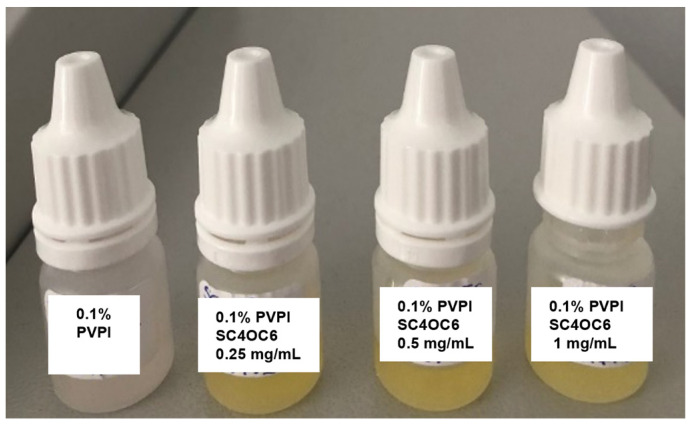
Samples of 0.1% PVPI solution alone (far left) and with different amounts of SC4OC6, after 3 months in plastic dropper containers at 4 °C.

The stabilizing effect of the SC4OC6 was even more distinct in samples stored in plastic dropper containers. In the 0.1% PVPI solution stored at room temperature, the triiodide absorption band in the UV-vis spectrum reduced to less than 1% after 21 days, whereas 40–50% of triiodide was retained in the presence of SC4OC6 (Figure S9). A longer stability was observed for samples stored in plastic containers at 4 °C. The picture in Figure 8 shows the faster discoloration of the PVPI solution without SC4OC6.

After 2 months at 4 °C, the retained triiodide absorbance was 10% for PVPI alone and 56%, 65%, and 90% in the presence of 0.25, 0.5, and 1 mg/mL of SC4OC6, respectively. After 3 months at 4 °C, the retained triiodide absorbance was 5% for PVPI alone and 32%, 57%, and 82% with 0.25, 0.5, and 1 mg/mL of SC4OC6, respectively (Table S2).

Determination of Iodine Amount

**Figure 9:**
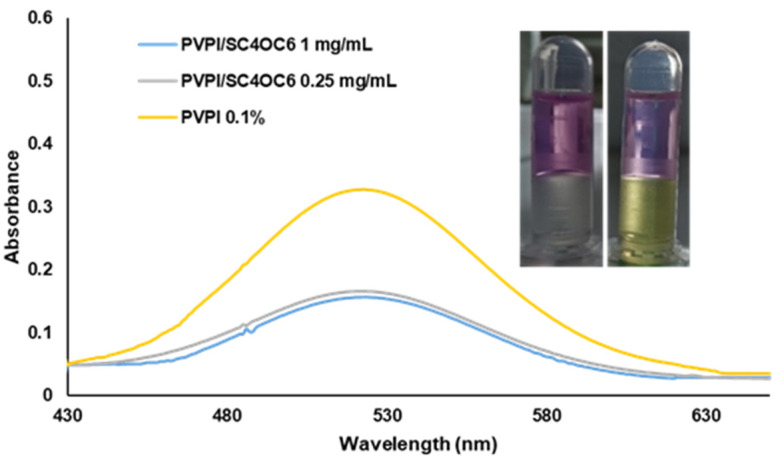
UV-vis spectra of iodine in cyclohexane (10 s shaking) from PVPI alone and with different amounts of SC4OC6; inset: pictures of the iodine extraction from a solution of 0.1% PVPI (left) and PVPI/SC4OC6 (right).

The lower loss of iodine from the 0.1% PVPI solution in the presence of SC4OC6 could be related to a lower amount of uncomplexed free iodine (I_2_) in solution. To support this assumption, the amount of free iodine was determined in the absence and presence of SC4OC6 by extraction with an organic solvent such as cyclohexane. It is known that organic solvents extract molecular iodine (I_2_) but not the water-soluble anionic iodide species, and the diffusion of I_2_ toward the organic solvent further moves iodine from the triiodide reserve. Exhaustive extraction of iodine with cyclohexane (1 mL × 3 times) from the PVPI and PVPI/SC4OC6 solutions provided an amount of available iodine (reservoir iodine plus free iodine) of around 100 ppm in both the samples. This value was calculated from the absorption at 522 nm (referred to a proper calibration curve) and it is consistent with the literature reporting that the available iodine ranges from 8.5% to 12% (USP standard) of the PVPI concentration. When a softer iodine extraction was performed by a rapid shaking of the solution (20 s, 1 mL cyclohexane), a lower amount of free iodine was present in the PVPI solution containing SC4OC6 (Figure 9). The amount of iodine in cyclohexane was around 80 ppm from the solution of PVPI alone and 40 ppm from the PVPI/SC4OC6 solution. The different amount of free iodine in the absence and presence of SC4OC6 was visible to the naked eye by the color of the aqueous and organic phases (Figure 9 inset).

**Figure 10:**
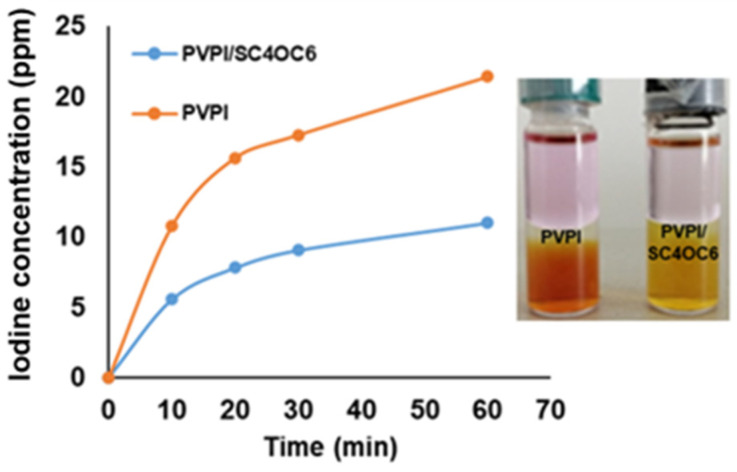
Diffusion of molecular iodine from 0.1% PVPI buffered solution (1 mL) to cyclohexane (1 mL) in the absence and in the presence of SC4OC6 (0.25 mg/mL) at different time intervals (5, 10, 20, 30, and 60 min); inset: picture of the samples after 24 h.

In a PVPI solution, the iodine responsible for the germicidal action is the molecular iodine in equilibrium at the time of use, and the diffusion of the molecular iodine toward an organic solvent can simulate the consumption by microbial and organic load [42]. With this in mind, we decided to monitor the diffusion of the molecular iodine from the PVPI 0.1% and PVPI/SC4OC6 (0.25 mg/mL) buffered solutions to cyclohexane. To this aim, cyclohexane was layered over the buffered solutions and the amount of molecular iodine in the cyclohexane phase was determined at fixed time intervals (10, 20, 30, and 60 min). The plot in Figure 10 shows that the iodine concentration in cyclohexane reduced by around 50% in the presence of SC4OC6.

This evidence further highlighted that the SC4OC6/PVPI nanoassembly enhances the iodine retention consistently with the higher stability of the PVPI/SC4OC6 solution compared to those containing PVPI alone.

### 3.4. Antibacterial Activity 

**Figure 11:**
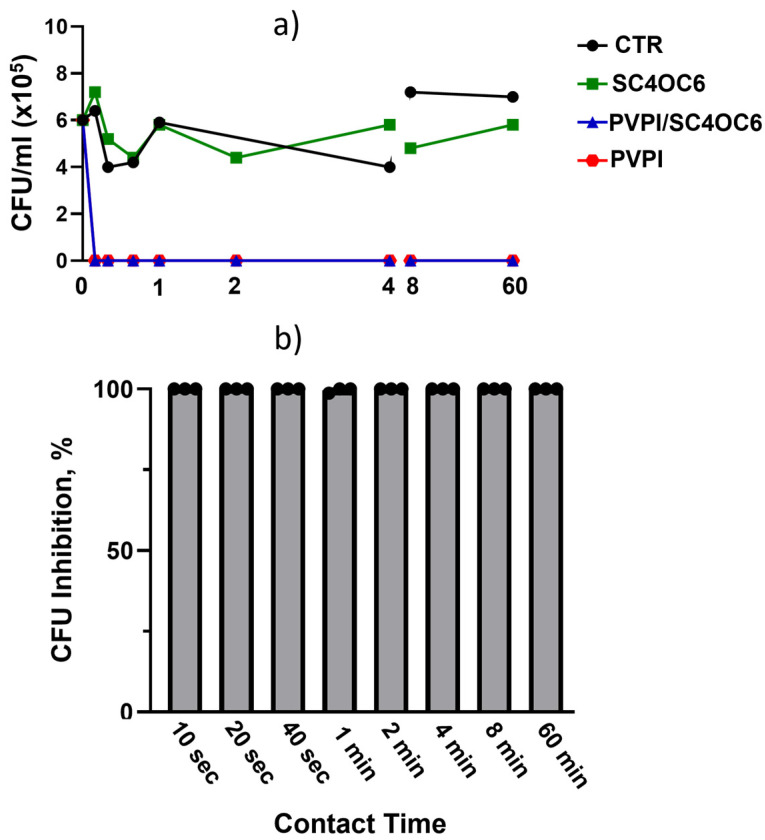
(**a**) Representative time-killing experiment performed against *Staphylococcus Epidermidis* for the vehicle PBS (CTR), PVPI 0.1%, SC4OC6 (0.25 mg/mL), and PVPI/SC4OC6; (**b**) inhibition (%) of the bacterial cell viability (3 time-killing experiments) by PVPI/SC4OC6. Statistical analysis (Two-Way ANOVA) of time-killing assay for PVPI/SC4OC6 vs. SC4OC6: 10, 20, and 40 s, 1 and 2 min, *p* < 0.01; 4 and 8 min *p* < 0.001; 60 min *p* < 0.05.

PVPI products are highly efficacious in vitro (>99.99% kill rate) against a wide range of clinically relevant bacterial and fungal pathogens [43]. PVPI has a rapid (within 30–60 s), complete bactericidal activity at both high and low concentration [43]. To verify if the bactericidal action of the PVPI is preserved in the presence of SC4OC6, we performed time-killing assays against a *Staphylococcus epidermidis* strain sensitive to PVPI [44]. The results showed that the presence of SC4OC6 does not affect the antibacterial activity of the PVPI. A 99.999% inhibition of the bacterial cell vitality (corresponding to a CFU/mL reduction >5 Log10) was observed for both PVPI and PVPI/SC4OC6 as early as 10 s after the contact time (Figure 11).

## 4. Conclusions

In this study, we demonstrated that a *p*-sulfonato-calix[4]arene amphiphile (SC4OC6) spontaneously forms polyanionic nanoaggregates in PCB. The micelles establish non-covalent interactions with PVPI as evidenced by UV-vis, NMR, and ITC analyses. ITC measurements revealed that the calix[4]arene scaffold and the micellar structure play a crucial role in the effective interaction with PVPI, as evidenced by the comparison with non-macrocyclic SDS micelles and non-micellar *p*-sulfonato-calix[4]arene receptors. Remarkably, the PVPI/SC4OC6 nanoassembly enhances the stability of a 0.1% PVPI buffered solution by retaining volatile iodine. Antibacterial assays performed against *S. epidermidis* evidenced that SC4OC6 does not perturb the ability of PVPI to eradicate bacterial growth rapidly and effectively.

Due to the toxicity of iodine, PVPI solutions at small concentrations are appealing tools for obtaining safer disinfectants/antiseptics for routine use, relevant in an era in which bacteria and viruses represent a serious threat. As a general antiseptic agent with broad-spectrum antimicrobial activity, the PVPI/SC4OC6 nanoformulation might substantially improve PVPI performances by reducing its toxicity risks.

## Figures and Tables

**Table 1:** Critical aggregation concentration (CAC) and enthalpy of aggregation (∆H) values for the povidone iodide (PVPI) self-aggregation process in different media at 25 °C.

	PVPI	PVPI/SC4OC6	PVPI/SDS	PVPI/SC4OH
CAC (mg mL^−1^)	0.426 (9)	0.117 (3)	0.14 (1)	n.d.
Δ*H* (kJ mol^−1^)	−4.4 (1)	−8.6 (4)	−4.3 (2)	n.d

## Data Availability

Data are available from the corresponding authors upon reasonable request.

## Supplementary Materials

The following supporting information can be downloaded at: https://www.mdpi.com/article/10.3390/nano13020286/s1, Figure S1: ITC titration of SC4OC6 1.1 mM into phosphate/citrate buffer 10 mM, pH 6, at 25 °C; Figure S2: ITC titration of SDS 10 mM into phosphate/citrate buffer 10 mM, pH 6, at 25 °C; Figure S3: Dynamic light scattering: size distribution by volume % of SC4OC6 in phosphate/citrate buffer (pH 6); Figure S4: UV-vis spectra of PVPI with SDS (blue) and SC4OC6 (0.25 mg/mL, orange); Figure S5: Dynamic light scattering: size distribution by intensity % of PVPI 0.1% alone (a) and with 0.25 mg/mL SC4OC6 (b) in phosphate/citrate buffer (pH 6); Figure S6: ITC titration of a 2.5 mg/mL PVPI solution into SC4OH 1 mM (phosphate/citrate buffer 10 mM, pH 6) at 25 °C (top); enthalpy of reaction as a function of the total concentration of PVPI (CPVPI calculated as the monomer unit) in the calorimetric cell (bottom); Figure S7: ITC titration of a 2.5 mg/mL PVPI solution into SDS 1 mM (phosphate/citrate buffer 10 mM, pH 6) at 25 °C (top); enthalpy of reaction as a function of the total concentration of PVPI in the calorimetric cell (CPVPI calculated as the monomer unit; squares: enthalpy values, red line: curve fitting, black line: first derivative) (bottom).; Figure S8: Thermodynamic parameters for the binding process of PVPI with SC4OC6 micelles at 25 °C and pH 6.; Figure S9: Absorption spectra of 0.1% PVPI (phosphate/citrate buffer, pH 6) alone (blue) and with SC4OC6 (0.25, 0.5, 1 mg/mL) after 21 days of storage in plastic drop containers; Table S1: CMC and enthalpy values for SC4OC6 and SDS self-aggregation process (phosphate/citrate buffer 10 mM, pH 6, 25 °C); Table S2: Retention of triiodide (absorbance %) at 4 °C.

## Abbreviations

PVPI	Polyvinylpyrrolidone iodine
SC4OES	*p*-sulfonato-calix[4]arene-O-hexyl
SC4OH	*p*-sulfonato-calix[4]arene
SDS	Sodium dodecyl sulfate
PCB	Phosphate-citrate buffer
PBS	Phosphate-buffered saline
CTR	vehicle PBS (control)
