# Supplementary material for: p-Sulfonato-Calix[4]arene Micelles Stabilize a Povidone Iodine Solution: Supramolecular Interactions, Iodine Retention, and Bactericidal Activity
Source: Nanomaterials (Basel). 2023 Jan 10;13(2):286. doi: 10.3390/nano13020286 (PMC9865561; doi:10.3390/nano13020286)
Supplement: Supplementary file 1 [file nanomaterials-13-00286-s001.zip › nanomaterials-2120873-supplementary.pdf]

nanomaterials

MDPI

Supplementary Materials

p-Sulfonato-Calix[4]arene Micelles Stabilize a Povidone Iodine Solution: Supramolecular Interactions, Iodine Retention, and Bactericidal Activity

Rossella Migliore [1,†], Loredana Ferreri [1,†], Danilo Aleo [2], Claudia Giovanna Leotta [3], Giovanni Mario Pitari [3], Nicola D'Antona [1], Carmelo Sgarlata [4,*] and Grazia Maria Letizia Consoli [1,*]

[1] Institute of Biomolecular Chemistry—National Research Council (C.N.R.), Via Paolo Gaifami 18, 95126 Catania, Italy
[2] MEDIVIS Srl, Via Carnazza 34C, Tremestieri Etneo, 95030 Catania, Italy
[3] Vera Salus Ricerca, Via Sigmund Freud 62/B, 96100 Siracusa, Italy
[4] Department of Chemical Sciences, University of Catania, Viale Andrea Doria 6, 95125 Catania, Italy
* Correspondence: sgarlata@unict.it (C.S.); grazia.consoli@icb.cnr.it (G.M.L.C.)
† These authors contributed equally to this work.

CONTENT

Figure S1. ITC titration of SC4OC6
Figure S2. ITC titration of SDS
Table S1. CMC and enthalpy values for SC4OC6 and SDS self-aggregation process
Figure S3. DLS of SC4OC6
Figure S4. UV-vis spectra of PVPI with SDS and SC4OC6
Figure S5. DLS of PVPI 0.1 % alone and with SC4OC6
Figure S6. ITC titration of a PVPI solution into SC4OH and enthalpy of reaction
Figure S7. ITC titration of a PVPI solution into SDS and enthalpy of reaction
Figure S8. Thermodynamic parameters for the binding process of PVPI with SC4OC6 micelles
Figure S9. Absorption spectra of 0.1% PVPI alone and with SC4OC6 after 21 days of storage
Table S2. Retention of triiodide (absorbance %) at 4 °C

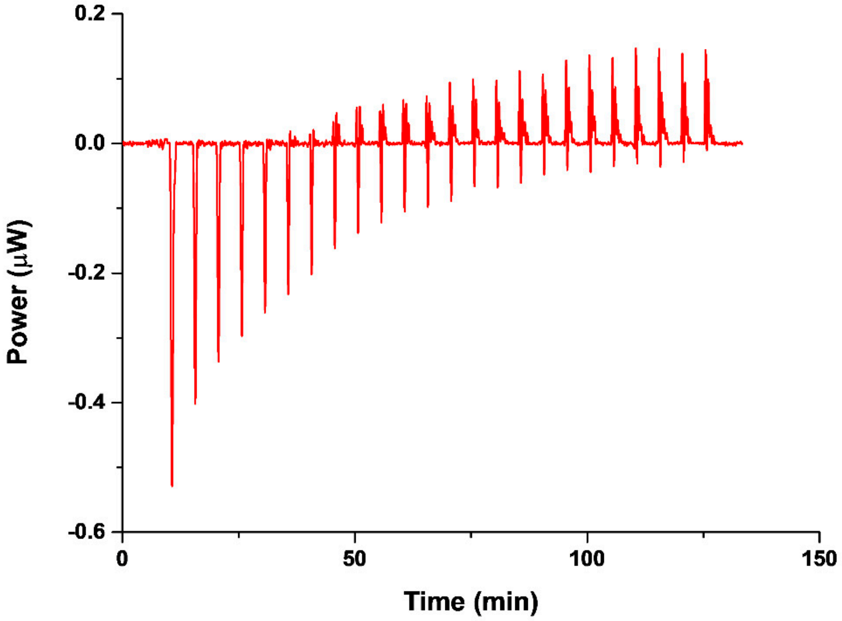

Figure S1. ITC titration of SC4OC6 1.1 mM into phosphate/citrate buffer 10 mM, pH 6, at 25 °C.

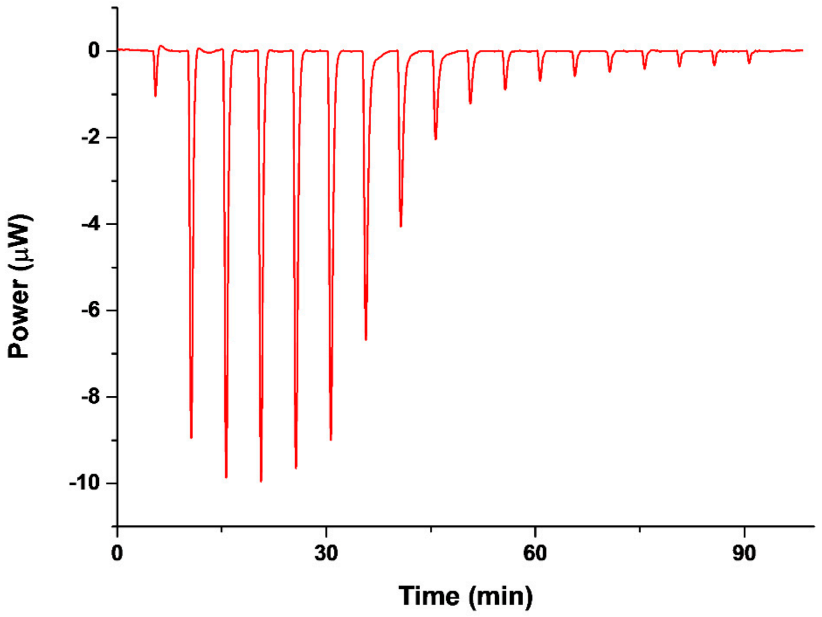

Figure S2. ITC titration of SDS 10 mM into phosphate/citrate buffer 10 mM, pH 6, at 25 °C.

Table S1. CMC and enthalpy values for SC4OC6 and SDS self-aggregation process (phosphate/citrate buffer 10 mM, pH 6, 25 °C).

	**CMC (mM)**	**ΔH (kJ mol⁻¹)**
SC4OC6	0.05 (2)	–1.7 (1)
SDS	0.76 (4)	–2.9 (2)

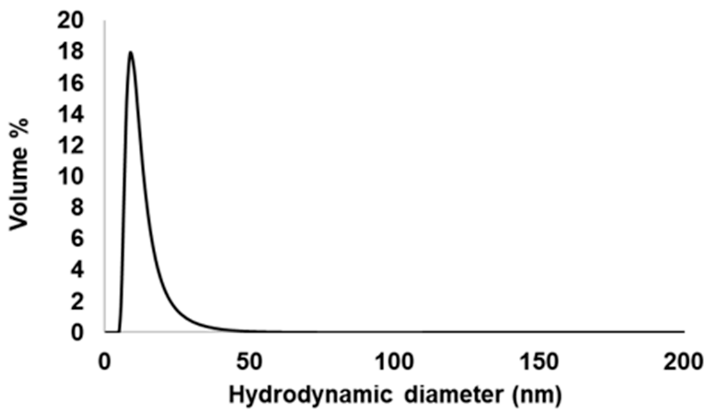

Figure S3. Dynamic light scattering: size distribution by volume % of SC4OC6 in phosphate/citrate buffer (pH 6).

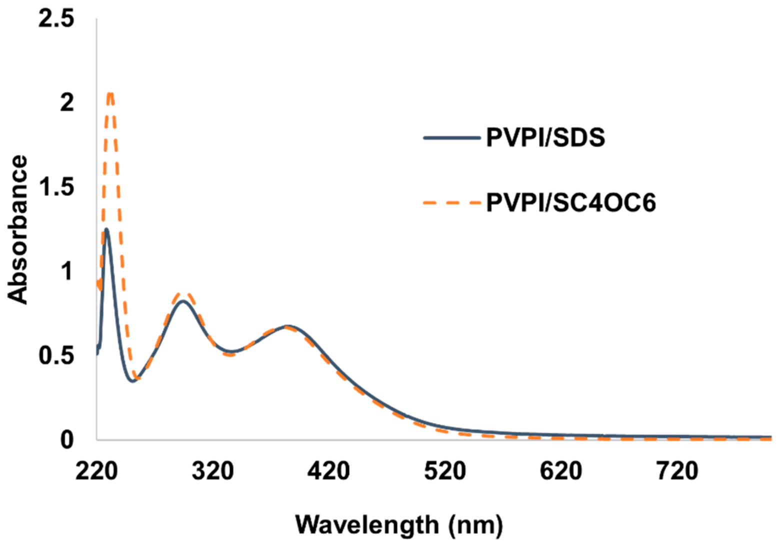

Figure S4. UV-vis spectra of PVPI with SDS (blue) and SC4OC6 (0.25 mg/mL, orange).

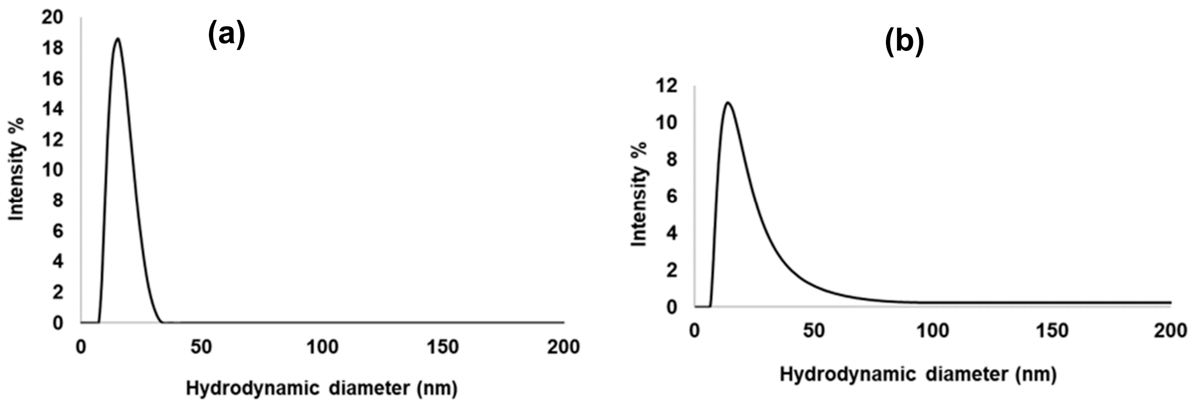

Figure S5. Dynamic light scattering: size distribution by intensity % of PVPI 0.1% alone (**a**) and with 0.25 mg/mL SC4OC6 (**b**) in phosphate/citrate buffer (pH 6).

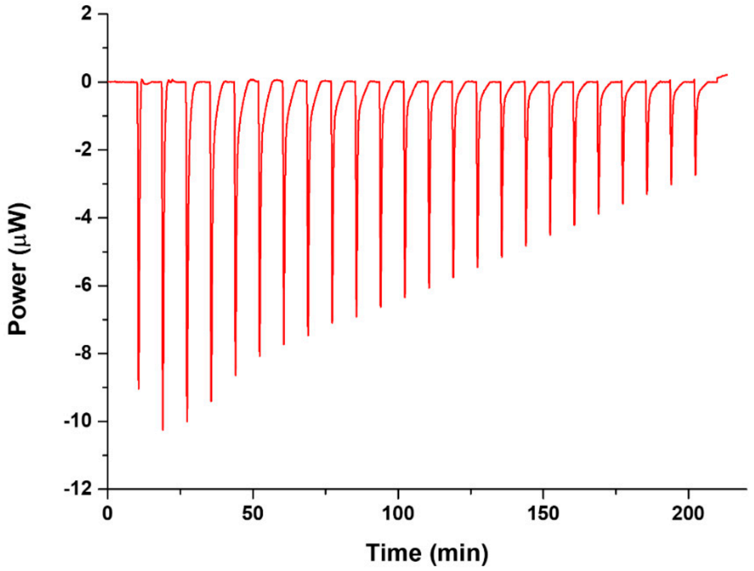

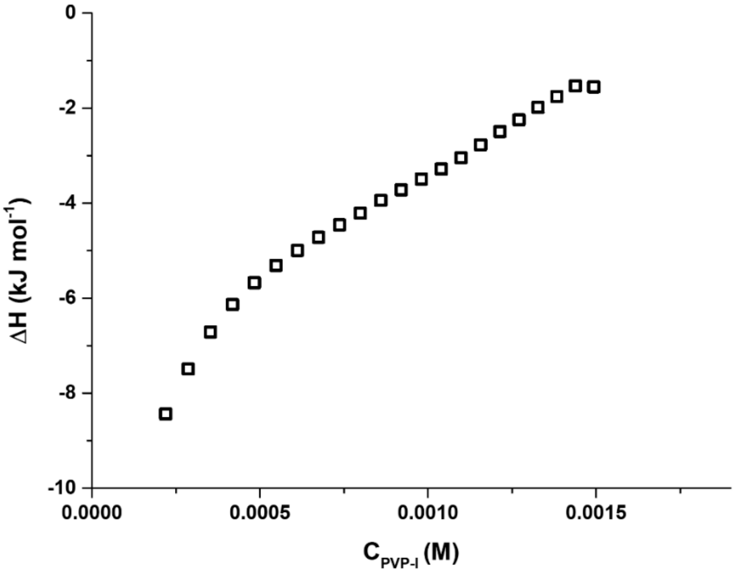

Figure S6. ITC titration of a 2.5 mg/mL PVPI solution into SC4OH 1 mM (phosphate/citrate buffer 10 mM, pH 6) at 25 °C (**top**); enthalpy of reaction as a function of the total concentration of PVPI (C_{PVPI} calculated as the monomer unit) in the calorimetric cell (**bottom**).

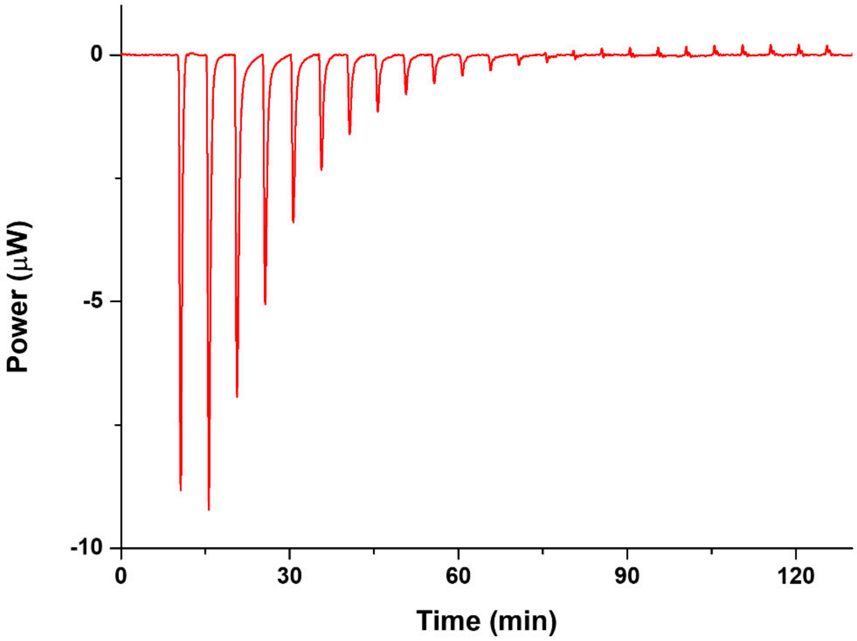

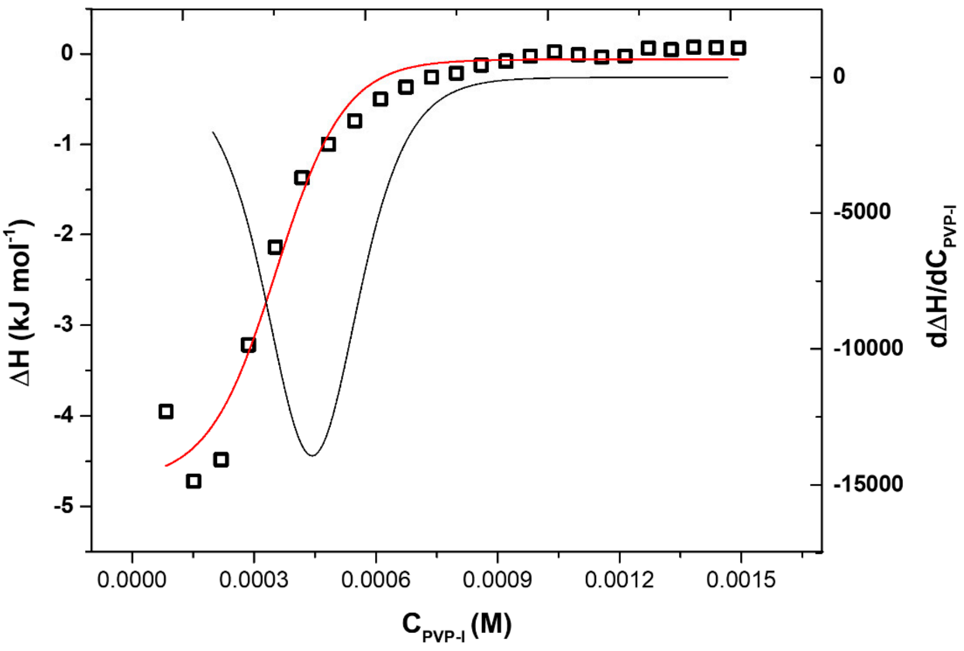

Figure S7. ITC titration of a 2.5 mg/mL PVPI solution into SDS 1 mM (phosphate/citrate buffer 10 mM, pH 6) at 25 °C (**top**); enthalpy of reaction as a function of the total concentration of PVPI in the calorimetric cell (C_{PVPI} calculated as the monomer unit; squares: enthalpy values, red line: curve fitting, black line: first derivative) (**bottom**).

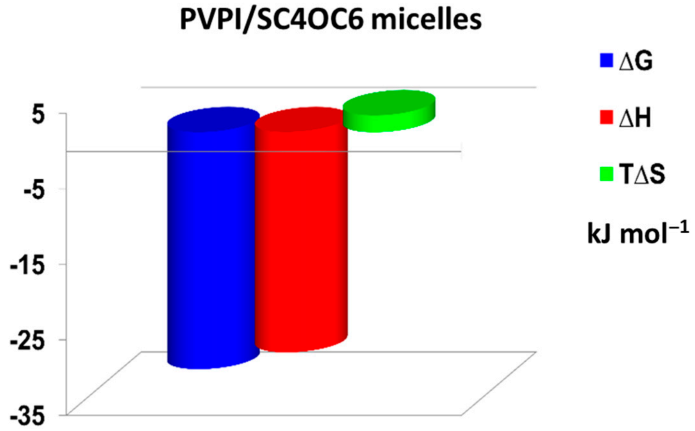

Figure S8. Thermodynamic parameters for the binding process of PVPI with SC4OC6 micelles at 25 °C and pH 6.

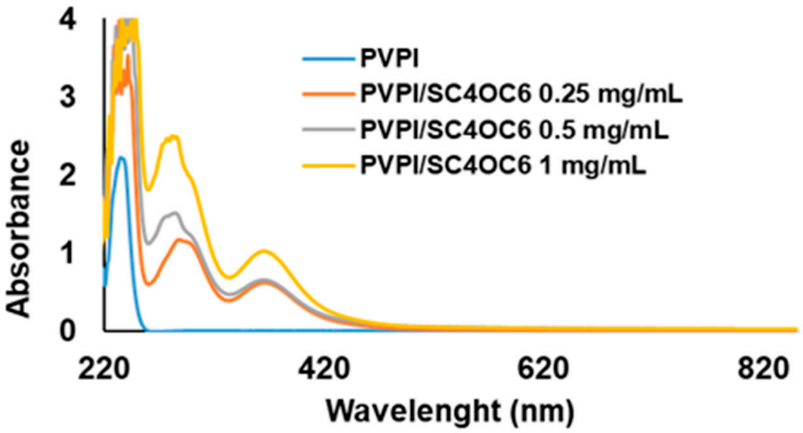

Figure S9. Absorption spectra of 0.1% PVPI (phosphate/citrate buffer, pH 6) alone (blue) and with SC4OC6 (0.25, 0.5, 1 mg/mL) after 21 days of storage in plastic drop containers.

Table S2. Retention of triiodide (absorbance %) at 4 °C.

Sample at 4 °C	Triiodide Absorbance Retention	
	2 Months	3 Months
PVPI 0.1%	10%	5%
PVPI 0.1%/SC4OC6 0.25 mg/mL	56%	32%
PVPI 0.1%/SC4OC6 0.5 mg/mL	65%	57%
PVPI 0.1%/SC4OC6 1 mg/mL	90%	82%
